# Characteristics and prognosis of electrical storm during acute myocardial infarction

**DOI:** 10.1093/europace/euaf077

**Published:** 2025-04-02

**Authors:** Alexandre Salaun, Raphael Martins, Sandro Ninni, Rayan Mohammed, Ruxandra Sava, Donovan Decaudin, Pierre Groussin, Dominique Pavin, François Brigadeau, Amine Tazibet, Soundous M’Rabet, Gabriel Laurent, Didier Klug, Antoine Da Costa, Karim Benali, Charles Guenancia

**Affiliations:** Department of Cardiology, Dijon University Hospital, 21000 Dijon, France; Section of Cardiac Electrophysiology, Rennes University, Rennes, France; INSERM-LTSI, U1099 Rennes, France; Heart and Lung Institute, Lille University, Lille, France; Section of Cardiac Electrophysiology, Saint-Etienne University, Saint-Etienne, France; Section of Cardiac Electrophysiology, Saint-Etienne University, Saint-Etienne, France; Section of Cardiac Electrophysiology, Rennes University, Rennes, France; INSERM-LTSI, U1099 Rennes, France; Section of Cardiac Electrophysiology, Rennes University, Rennes, France; INSERM-LTSI, U1099 Rennes, France; Section of Cardiac Electrophysiology, Rennes University, Rennes, France; Heart and Lung Institute, Lille University, Lille, France; Heart and Lung Institute, Lille University, Lille, France; Department of Cardiology, Dijon University Hospital, 21000 Dijon, France; PEC 2 EA 7460, UFR Sciences de Santé, Université de Bourgogne, 21000 Dijon, France; Department of Cardiology, Dijon University Hospital, 21000 Dijon, France; PEC 2 EA 7460, UFR Sciences de Santé, Université de Bourgogne, 21000 Dijon, France; Heart and Lung Institute, Lille University, Lille, France; Section of Cardiac Electrophysiology, Saint-Etienne University, Saint-Etienne, France; INSERM-LTSI, U1099 Rennes, France; Section of Cardiac Electrophysiology, Saint-Etienne University, Saint-Etienne, France; IHU LIRYC, Electrophysiology and Heart Modeling Institute, Bordeaux, France; Department of Cardiology, Dijon University Hospital, 21000 Dijon, France; PEC 2 EA 7460, UFR Sciences de Santé, Université de Bourgogne, 21000 Dijon, France

**Keywords:** Electrical storm, Ventricular fibrillation, Ventricular tachycardia, Acute myocardial infarction, PAINESD score, STEMI, NSTEMI, Prognosis

## Introduction

Electrical storm (ES) is a severe cardiac event characterized by at least three episodes of sustained ventricular arrhythmias (VAs) within 24 h or incessant VA for >12 h. It significantly increases mortality, particularly in acute myocardial infarction (AMI). Despite advancements in AMI management, including widespread use of percutaneous coronary intervention (PCI), ventricular tachycardia (VT), and ventricular fibrillation (VF) remain major complications.^1^ The incidence of VA in AMI varies, with estimates of 6–10% in ST-segment elevation myocardial infarction (STEMI) and 2.1% in non-STEMI (NSTEMI). Electrical storm is the most severe form, with a reported mortality of 22–54%.^2,3^

Although studies have investigated unclustered VA in AMI, data specifically addressing ES remain scarce. Understanding ES characteristics, prognostic factors, and clinical outcomes is essential for improving risk stratification and treatment strategies. Additionally, the PAINESD score, originally developed to predict haemodynamic decompensation in VT ablation candidates, may help identify high-risk AMI patients with ES.^4^ This study aimed to assess the characteristics and prognosis of ES in AMI, identify factors associated with in-hospital mortality, and evaluate the PAINESD score’s predictive value.

## Methods

### Study population

This retrospective, multi-centre study included all patients with ES during AMI from a cohort of 807 patients admitted to four French tertiary centres between 2010 and 2023. An ES was defined as three or more sustained VT/VF episodes within 24 h, incessant VA for >12 h, or three or more appropriate implantable cardioverter defibrillator (ICD) therapies within 24 h.

### PAINESD score

The PAINESD score consists of eight clinical variables: chronic obstructive pulmonary disease, age >60 years, ischaemic cardiomyopathy, New York Heart Association (NYHA) Class III/IV, left ventricular ejection fraction (LVEF) <25%, ES occurrence, diabetes, and prior VT ablation.^4^

### Statistical analysis

Continuous variables were analysed using *t*-tests or Mann–Whitney *U* tests, while categorical data were compared using χ^2^ or Fisher’s exact tests. The optimal threshold for discriminating in-hospital mortality using PAINESD score was obtained with the receiver-operating characteristic curve with the best sensitivity and specificity according to the Youden index. The multivariate logistic regression model included non-collinear variables according to their univariate relationship with in-hospital mortality (*P* < 0.05).

## Results

### Patient characteristics

Among the 151 patients with ES during AMI, 85% were male, with a mean age of 61.5 years. Most had STEMI (78%), and the left anterior descending artery was the most frequent culprit lesion (45%). Mean LVEF was 32%, and 38% had previous ischaemic cardiomyopathy (*Table 1*).

**Table 1:** Baseline characteristics, characteristics, and management of the electrical storm

	All (*n* = 151)	Alive at discharge (*n* = 109)	In-hospital deaths (*n* = 42)	*P*-value
**Demographic characteristics**				
** **Gender (female)	23 (15.2%)	14 (12.8%)	9 (21.4%)	0.188
Age (years)	61.5 ± 12.2	61 ± 11.8	62.8 ± 13.3	0.413
Weight (kg)	79.6 ± 16.8	79.4 ± 16.9	80.3 ± 16.9	0.792
Height (cm)	172 ± 7.9	172 ± 7.6	172 ± 8.8	0.980
Body mass index (kg/m^2^)	26.6 ± 4.9	26.8 ± 5.3	26.3 ± 3.8	0.873
**Medical history**				
LVEF (%)	32.5 ± 14.2	33.7 ± 13.6	29.3 ± 15.3	0.094
Ischaemic cardiomyopathy	57 (37.7%)	48 (44%)	9 (21.4%)	**0**.**010**
AMI	52 (34.4%)	44 (40.4%)	8 (19%)	**0**.**013**
NYHA				**0.002**
I	50 (36.2%)	43 (44.3%)	7 (17.1%)	
II	44 (31.9%)	32 (33%)	12 (29.3%)	
III	17 (12.3%)	8 (8.2%)	9 (22.0%)	
IV	27 (19.6%)	14 (14.4%)	13 (31.7%)	
Cardiac surgery	7 (6.1%)	4 (4.9%)	3 (9.1%)	0.407
Atrial fibrillation	19 (12.6%)	13 (11.9%)	6 (14.3%)	0.695
Hypertension	76 (50.3%)	49 (45%)	27 (64.3%)	**0**.**033**
Diabetes mellitus	41 (27.2%)	27 (24.8%)	14 (33.3%)	0.289
Dyslipidaemia	61 (40.4%)	43 (39.4%)	18 (42.9%)	0.702
Smoking	83 (55%)	58 (53.2%)	25 (59.5%)	0.485
Peripheral artery disease	8 (5.3%)	5 (4.6%)	3 (7.1%)	0.686
COPD	6 (5.1%)	2 (2.4%)	4 (12.1%)	0.052
Stroke/TIA	8 (5.3%)	7 (6.4%)	1 (2.4%)	0.444
Chronic kidney failure	8 (5.3%)	6 (5.5%)	2 (4.8%)	1.000
Prior VAs	13 (8.6%)	11 (10.1%)	2 (4.8%)	0.517
Prior VF	4 (2.7%)	4 (3.7%)	0 (0%)	0.577
Prior VT	7 (4.7%)	6 (5.6%)	1 (2.4%)	0.674
Prior ES	3 (2%)	2 (1.9%)	1 (2.4%)	1.000
Prior ICD	14 (9.3%)	10 (9.2%)	4 (9.5%)	1.000
PAINESD score	16.3 ± 4.3	15.4 ± 3.9	18.7 ± 4.4	**<0**.**001**
**Biology**				
Creatinine (µmol/L)	88.2 ± 5.5	80 ± 51.6	108 ± 94.5	0.071
Kaliaemia (mEq/L)	4.3 ± 0.06	4.2 ± 0.66	4.3 ± 0.74	0.324
**Drug therapy at baseline**				
** **Amiodarone	10 (6.7%)	7 (6.5%)	3 (7.1%)	1.000
Beta-blockers	49 (32.7%)	38 (35.2%)	11 (26.2%)	0.292
**Aetiology**				
STEMI	118 (78.1%)	84 (77.1%)	34 (81%)	0.604
NSTEMI	33 (21.9%)	25 (22.9%)	8 (19%)	0.604
Time between onset of symptoms and revascularization (min)	5082 ± 8089	5155 ± 8542	4854 ± 6608	0.860
Duration of angioplasty (min)	69.7 ± 41.4	57.8 ± 31.6	83.3 ± 61.3	0.095
Culprit lesion				0.649
Left anterior descending artery	66 (44.6%)	50 (45.9%)	16 (41%)	
Circumflex artery	13 (8.8%)	11 (10.1%)	2 (5.1%)	
Right coronary artery	31 (20.9%)	21 (19.3%)	10 (25.6%)	
Left main artery or multi-vessel disease	38 (25.7%)	27 (24.8%)	11 (28.2%)	
TIMI grade before revascularization				0.465
0	88 (76.5%)	63 (76.8%)	25 (75.8%)	
1	12 (10.4%)	7 (8.5%)	5 (15.2%)	
2	2 (1.7%)	1 (1.2%)	1 (3.0%)	
3	13 (11.3)	11 (13.4%)	2 (6.1%)	
TIMI grade after revascularization				0.736
0	18 (15.3%)	13 (15.5%)	5 (14.7%)	
1	4 (3.4%)	2 (2.4%)	2 (5.9%)	
2	5 (4.2%)	3 (3.6%)	2 (5.9%)	
3	91 (77.1%)	66 (78.6%)	25 (73.5%)	
Time from AMI to ES onset (days)	5.9 ± 10.5	6.4 ± 11.6	4.4 ± 6.5	0.156
Time from AMI to ES onset >48 h	69 (50.7%)	53 (53%)	16 (43%)	0.191
**Type of VA**				
Monomorphic VT	57 (38%)	43 (40.6%)	14 (35%)	0.539
Polymorphic VT	19 (13%)	11 (10.4%)	8 (20%)	0.123
Torsade de pointes	9 (6.1%)	7 (6.5%)	2 (5%)	1.000
VF	99 (67.8%)	70 (66%)	29 (72.5%)	0.456
**Initial cause of hospitalization**				
ES	50 (33.1%)	40 (36.7%)	10 (23.8%)	0.132
Acute coronary syndrome	103 (68.2%)	78 (71.6%)	25 (59.5%)	0.155
Cardiac arrest	33 (21.9%)	24 (22%)	9 (21.4%)	0.937
Heart failure/cardiogenic shock	43 (28.5%)	25 (22.9%)	18 (42.9%)	**0**.**015**
**Haemodynamic tolerance of the ES**				
Pulmonary oedema/cardiogenic shock	53 (35.1%)	30 (27.5%)	23 (54.8%)	**0**.**002**
Refractory cardiac arrest	42 (27.8%)	25 (22.9%)	17 (40.5%)	**0**.**031**
**Management of the ES**				
Electrical cardioversion without ATP	101 (66.9%)	71 (65.1%)	30 (71.4%)	0.462
Electrical cardioversion + ATP	9 (6%)	6 (5.5%)	3 (7.1%)	0.709
Coronary angiography	144 (95.4%)	105 (96.3%)	39 (92.9%)	0.363
Revascularization performed	108 (71.5%)	79 (72.5%)	29 (69%)	0.676
Complete revascularization	36 (27.7%)	28 (30.8%)	8 (20.5%)	0.231
Light sedation	40 (26.5%)	24 (22%)	16 (38.1%)	**0**.**045**
Amiodarone	131 (87.9%)	95 (88%)	36 (87.8%)	0.979
Lidocaine	88 (59.1%)	58 (53.7%)	30 (73.2%)	**0**.**031**
Vasopressive drugs (amines)	80 (53%)	50 (45.9%)	30 (71.4%)	**0**.**005**
Intra-aortic balloon pump	21 (13.9%)	13 (11.9%)	8 (19%)	0.257
Impella	9 (6%)	4 (3.7%)	5 (11.9%)	0.116
ECLS	44 (29.1%)	23 (21.1%)	21 (50%)	**<0.001**
Dialysis	11 (7.3%)	6 (5.5%)	5 (11.9%)	0.175
Deep sedation	89 (58.9%)	54 (49.5%)	35 (83.3%)	**<0.001**
Radiofrequency catheter ablation	30 (19.9%)	24 (22%)	6 (14.3%)	0.286
Stellate ganglion block	11 (7.3%)	7 (6.5%)	4 (9.5%)	0.502

The values are expressed as *n* (%) and mean (±standard deviation). Bold indicates *P* < 0.05.

AMI, acute myocardial infarction; ATP, anti-tachycardia pacing; COPD, chronic obstructive pulmonary disease; ECLS, extracorporeal life support; ES, electrical storm; ICD, implantable cardioverter defibrillator; LVEF, left ventricular ejection fraction; NSTEMI, non-ST-segment elevation myocardial infarction; NYHA, New York Heart Association class of dyspnoea; STEMI, ST-segment elevation myocardial infarction; TIA, transient ischaemic attack; TIMI, thrombolysis in myocardial infarction score; VA, ventricular arrhythmia; VF, ventricular fibrillation; VT, ventricular tachycardia.

Hypertension was significantly associated with in-hospital mortality (64 vs. 45%, *P* = 0.033), as was NYHA class on admission (*P* = 0.002). The PAINESD score was significantly associated with in-hospital mortality, with a median value of 14 (11–17) in the group of patients alive at discharge and 20 (14–23) in the group of patients who died in the hospital (*P* < 0.001). However, AMI type (STEMI vs. NSTEMI), culprit artery, and thrombolysis in myocardial infarction (TIMI) flow grades before and after revascularization were not associated with mortality.

### Management and haemodynamic impact

Patients with ES had high rates of cardiogenic shock (35%) and refractory cardiac arrest (27.8%), both correlating with mortality (*P* = 0.002 and *P* = 0.031, respectively). Use of inotropic support (71 vs. 46%, *P* = 0.005) and extracorporeal life support (50 vs. 21%, *P* < 0.001) was higher in non-survivors.

There was no difference in mortality between VT- and VF-predominant ES. Electrical cardioversion, anti-arrhythmic therapy (amiodarone and lidocaine), and catheter ablation were used in both groups, with radiofrequency ablation performed in 20% of cases.

### Predictors of in-hospital mortality

Multivariate analysis identified NYHA Class >2 [odds ratio (OR) = 3.66, 95% confidence interval (CI): 1.61–8.32, *P* = 0.002] and previous hypertension (OR = 2.87, 95% CI: 1.25–6.57, *P* = 0.013) as independent mortality predictors. The PAINESD score had an area under the characteristic (AUC) curve of 0.717 (95% CI: 0.622–0.812), with a cut-off of ≥18, yielding 59.5% sensitivity and 81.7% specificity for predicting mortality.

### Post-discharge outcomes

Of the 109 patients discharged alive after a median follow-up of 272 (13–981) days, 14 (13%) had recurrent VA (of which 10 were unclassified VT/VF and 2 had recurrent ES) and 10 (9%) died (4 from end-stage heart failure, 1 from a non-cardiac cause, and 5 from an unknown cause).

## Discussion

This is the largest study analysing ES in AMI, showing a high in-hospital mortality rate of 28%. Our findings confirm that NYHA Class >2 and hypertension are key mortality predictors, emphasizing the role of pre-existing cardiac dysfunction in poor outcomes.

Unlike prior studies suggesting differential mortality risks based on AMI type, our results indicate STEMI and NSTEMI patients with ES have comparable in-hospital outcomes.^5^ Similarly, neither culprit artery distribution nor TIMI flow restoration was associated with mortality, suggesting factors beyond revascularization influence prognosis. These findings align with studies showing that despite PCI, residual ischaemic burden and electrical instability contribute significantly to mortality.^6^

The PAINESD score, originally developed for VT ablation risk stratification, showed moderate predictive accuracy (AUC = 0.717) in identifying patients with high-risk ES. A threshold of ≥18 differentiated survivors from non-survivors. This suggests that PAINESD scoring may help prioritize early intensive management, such as circulatory support or catheter ablation, for select patients.^7^

The high prevalence of haemodynamic instability in patients with ES (55% of non-survivors had cardiogenic shock) emphasizes the need for aggressive early intervention. Prior studies show that timely circulatory support improves ES survival, supporting its use in high-risk AMI cases.^8,9^

Additionally, our study highlights hypertension as a key ES mortality predictor, a factor often overlooked in AMI prognosis. Hypertension-induced microvascular dysfunction and delayed recognition of haemodynamic deterioration may contribute to poor outcomes.^10^ Future studies should assess whether earlier anti-hypertensive therapy in patients with ES could improve outcomes.

## Conclusions

Electrical storm during AMI is associated with high in-hospital mortality, primarily due to acute heart failure. The PAINESD score may be a useful tool for stratifying high-risk patients, with a cut-off ≥18 identifying those at greatest risk of death. Future prospective studies should validate PAINESD’s role in AMI-related ES and refine treatment strategies for these critically ill patients.

## Data Availability

Data supporting the findings of this study are available from the corresponding author upon reasonable request.
